# Clinical Profile and Treatment Outcomes in Patients Treated with Intensity-Modulated Radiotherapy (IMRT) for Carcinoma Nasopharynx: A Retrospective Analysis

**DOI:** 10.1155/2021/9932749

**Published:** 2021-08-30

**Authors:** Farida Nazeer, R. Rejnish Kumar, Malu Rafi, Tapesh Bhattacharya, Aparna Mullangath Prakasan, Kumar P. Naveen, Preethi George, Ramadas Kunnambath, Kainickal Cessal Thommachan

**Affiliations:** ^1^Department of Radiation Oncology, Regional Cancer Centre, Thiruvananthapuram, Kerala, India; ^2^Department of Cancer Epidemiology and Biostatistics, Regional Cancer Centre, Thiruvananthapuram, Kerala, India

## Abstract

**Objective:**

To retrospectively evaluate the clinical outcome of carcinoma nasopharynx patients treated with the IMRT technique.

**Methods:**

Eighty-one nasopharyngeal carcinoma patients who were treated with IMRT with or without chemotherapy between the period January 2011 and December 2014 at a comprehensive tertiary cancer center, Kerala, India, were included in the study. The mean age was 43 years (range 13–77 years), and majority of the patients were males (67.9%). The stagewise distribution of disease at presentation was 2 (2.5%) in stage I, 19 in stage II (23.5%), 31 (38.3%) in stage III, and 29 (35.8%) in stage IV. All patients were treated using simultaneous integrated boost (SIB) schedule using IMRT with 6 MV photon to a dose of 66 Gy in 30 fractions, 2.2 Gy per fraction prescribed to high-risk PTV; 60 Gy in 30 fractions, 2 Gy per fraction to intermediate risk PTV; and 54 Gy in 30 fractions, 1.8 Gy per fraction to low-risk PTV. Concurrent chemotherapy with cisplatin was offered to patients with stage II and above disease. Neoadjuvant chemotherapy with cisplatin and 5FU was given to patients with initially advanced disease (T3, T4, N2, and N3). Survival estimates were generated using the Kaplan–Meier method. The univariate analysis was performed using log-rank tests.

**Results:**

The 5-year locoregional control (LRC), distant metastasis-free survival (DMFS), disease-free survival (DFS), and overall survival (OS) rates were 87.5%, 87%, 61.6%, and 62.5%, respectively. The 5-year OS was 100% for stage I (*n* = 2), 67% for stage II (*n* = 19), 70.4% for stage III (*n* = 31), and 68.1% for stage IV (*n* = 29). The DFS at 5 years was 100% for stage I, 61.1% for stage II, 56.2% for stage III, and 84.8% for stage IV disease. The univariate analysis showed that age, nodal stage, and use of induction chemotherapy showed an improved trend towards OS, though the results were not statistically significant. The predominant pattern of failure in the present study was distant metastasis. Most patients who developed distant metastasis in our study had either an advanced *T* stage or N3 disease at presentation.

**Conclusion:**

The present study shows our initial experience with IMRT for nasopharyngeal carcinoma. The compliance to RT was good in this study. The 5-year LRC and OS rate of nasopharyngeal carcinoma patients treated with IMRT were 87.5% and 62.5%. Distant metastasis was the main pattern of failure.

## 1. Introduction

Concurrent chemoradiation with or without induction chemotherapy is the standard of care for locally advanced nasopharyngeal carcinoma [1, 2]. The dose delivered to the tumor determines the tumor control in nasopharyngeal carcinoma [3]. Initially, 2D and 3DCRT techniques were used for the treatment of nasopharyngeal carcinoma. Earlier studies have shown that with the 2D technique, the locoregional control for T1 and T2 tumors was excellent but was lower with T3 and T4 disease [4, 5]. Later on, with the development of intensity-modulated radiation therapy (IMRT), a higher dose delivery to the tumor was possible which increased locoregional control for even T3 and T4 tumors and increased overall survival with reduction of acute and late toxicities [6, 7]. A study by Peng et al., compared 2D with IMRT, has also shown the superiority of IMRT in terms of local control and overall survival [8]. Thus, IMRT became the standard of care in treatment of nasopharyngeal carcinoma. In this background, we did a retrospective analysis to study the clinical outcome of patients with nasopharyngeal carcinoma who were treated in the earlier years with the IMRT technique in our institution.

## 2. Methods and Materials

### 2.1. Patient Cohort

A retrospective analysis of eighty-one patients with biopsy-proven primary nasopharyngeal carcinoma who were treated with radiotherapy using the IMRT technique with or without chemotherapy between the period January 2011 and December 2014 at a comprehensive tertiary cancer center, Kerala, India, were analyzed in the study. Patients with metastatic disease at presentation, recurrent disease, and patients treated with palliative intent were excluded from the study.

All patients with biopsy-proven squamous cell carcinoma nasopharynx were staged using a standard protocol comprising of clinical examination, endoscopic assessment of primary, CT/MRI scan of the head and neck, and chest X-ray. Metastatic workup with ultrasound abdomen and bone scan was done for stages III and IV disease and for patients with undifferentiated histology. Staging of disease was carried out according to the International Union Against Cancer (UICC)/American Joint Committee on Cancer (AJCC) 2010 staging classification. All patients had a pretreatment dental evaluation and nutritional assessment. An EBV assay was not routinely carried out.

### 2.2. Treatment

All patients received radiotherapy (IMRT) using the linear accelerator with or without chemotherapy. All patients underwent CT simulation after proper immobilization.

CT images were imported onto a treatment planning system followed by delineation of target volumes and critical organs at risk. Gross tumor volume (GTV) primary was defined as all detectable gross tumors as seen on imaging studies, endoscopy, and clinical examination. GTV node was defined as any enlarged lymph nodes seen on imaging. The high-risk clinical target volume (CTV) primary (CTVp66) included 5 mm margin around GTVp. Similarly, CTVn66 included GTV node plus 5 mm margin or 10 mm margin in case of extranodal extension. This CTV 66 was edited from natural anatomical barriers such as the bone and air.

The intermediate risk CTV (CTVp60) included the CTVp1 plus 5 mm and those areas with high chance of harboring microscopic disease. It included the entire nasopharynx, anterior 1/2 to 2/3 of the clivus (entire clivus if clinically involved), inferior sphenoid sinus, skull base including bilateral foramen ovale and rotundum, pterygoid fossa, parapharyngeal space, and posterior one-third of nasal cavity and maxillary sinuses (to ensure pterygopalatine fossa coverage). The entire sphenoid sinus and cavernous sinus were included in T3 and T4 disease. The intermediate risk nodal CTV (CTVn60) included the bilateral levels II, III, and V, retrostyloid, and lateral retropharyngeal lymph nodes. If level III nodes were involved clinically, then level IV and supraclavicular lymph nodes were also included in CTV n2.

The low-risk CTV (CTV 54) included the bilateral uninvolved lower nodal levels. A 5 mm volumetric expansion was used to generate the planning target volumes PTV66, PTV60, and PTV54 from the corresponding CTVs.

The organ at risk (OAR) structures contoured included the parotid glands, brainstem, spinal cord, temporal lobe, cochlea, optic nerve, optic chiasm, eye, lens, pharyngeal constrictors, oral cavity, mandible, larynx, and brachial plexus. A 3 mm margin was given to the spinal cord and optic apparatus to create the planning organ at risk volume (PRV).

IMRT plans were generated using an inverse planning algorithm. Dose constraints for target volume were evaluated according to RTOG criteria and for OAR according to Quantitative Analysis of Normal Tissue Effects in the Clinic (QUANTEC) results.

### 2.3. Dose Prescription and Delivery

All patients were treated using simultaneous integrated boost (SIB) schedule using IMRT with 6 MV photon. A dose of 66 Gy in 30 fractions, 2.2 Gy per fraction was prescribed to PTV 66; 60 Gy in 30 fractions, 2 Gy per fraction to PTV 60; and 54 Gy in 30 fractions, 1.8 Gy per fraction to PTV 54. The patients were treated in once daily fractions, 5 days a week for a total duration of 6 weeks. Plans were optimized to deliver 100% dose to at least 95% of PTV, and less than 10% of PTV 66 receive more than or equal to 107%.

The maximum dose to critical normal structures were as follows: brain stem 54 Gy, spinal cord 45 Gy, optic apparatus 54 Gy, cochlea 45 Gy, lens 10 Gy, mandible 70 Gy, constrictors 50 Gy, larynx 45 Gy, brachial plexus 66 Gy, and oral cavity 40 Gy. The mean dose to one parotid was kept less than 26 Gy or 50% of the gland must receive less than 30 Gy.

Treatment was delivered using the Rapid Arc IMRT technique with the Varian Clinac IX linear accelerator with CBCT initially and then as and when required. A daily KV portal imaging was conducted to check for any set up errors.

### 2.4. Chemotherapy

Concurrent chemotherapy was offered to patients with stage II and above disease using cisplatin at a dose of 80–100 mg/m^2^ on D1 every three weeks. Neoadjuvant chemotherapy was given to patients with initial advanced primary and nodal disease (T3, T4, N2, and N3). Neoadjuvant chemotherapy was with PF regimen (cisplatin at a dose of 75–100 mg/m2 IV infusion on day 1 and 5-fluorouracil 750–1000 mg/m^2^ IV 24-hour infusion on days 1–4, every three weeks). Adjuvant chemotherapy (PF regimen) was offered to patients in our institution during the initial years. However, since 2012, we had stopped practicing adjuvant chemotherapy.

### 2.5. Follow-Up of Patients

All patients were reviewed weekly during radiotherapy. After completion of planned course of treatment, the patients were followed up at regular intervals which included an ENT evaluation. A reassessment CT scan of the head and neck was performed at four to six months posttreatment and thereafter as and when required. Serum TSH estimation was performed at 6 months posttreatment and thereafter at yearly intervals.

### 2.6. Statistics

The details of patients, their tumor and treatment-related characteristics, and late toxicities were retrieved from the hospital database using a structured proforma. The patients were followed up till March 30, 2018. The primary endpoints analyzed were disease-free survival (DFS) and overall survival (OS), and secondary end points were locoregional control (LRC) and distant metastasis-free survival (DMFS).

DFS was defined as the period from the date of registration to the date of locoregional relapse or distant relapse or the date of death whichever has occurred earlier. OS was defined as the period from the date of registration to the date of death due to any cause. LRC was defined as the period from the date of registration to date of relapse in primary or nodal or both sites and DMFS from the date of registration to the date of relapse in distant sites or death whichever had occurred first.

Survival estimates were generated using the Kaplan–Meier method. The univariate analysis would be performed using log-rank tests, and prognostic factors were planned to be assessed using the Cox proportional hazards regression model. For the univariate analysis, the outcome measures (LRC, DFS.OS, and DMFS) were correlated with various factors such as age, *T* stage, *N* stage, composite stage, and sequencing of chemotherapy. Patients had been stratified into two age groups (age <50 and age ≥ 50) for analysis of the outcomes. With respect to chemotherapy, the use of neoadjuvant, induction, and concurrent chemotherapy were separately analyzed for any significant association with outcomes.

## 3. Results

A total of eighty-one patients were included in the study, and the median follow-up period was 59 months. The mean age of the target population was 43 years (range 13–77 years), and majority of the patients were males (67.9%). The demographic and clinical characteristics of patients are given in Table 1. Majority of the patients had stage III disease at presentation (38.3%), and most of the patients had WHO type 2b histology (96.3%).

The details of chemotherapy received are given in Table 2. Forty-two patients had received neoadjuvant chemotherapy (10 patients had 1 cycle, 19 had 2 cycles, 12 had 3 cycles, and 1 had 4 cycles), and sixty-nine received concurrent chemotherapy of which 2 patients received only 1 cycle, 58 received 2 cycles, and 9 received 3 cycles. Seven patients have not received chemotherapy at all, of which two had stage 1 disease and the other five had advanced age and poor performance status.

All patients completed the planned course of radiotherapy without any interruption.

After radical treatment, 19 (23.5%) patients had relapsed. The median time to recurrence of disease was 12 months. The most common site of relapse was at distant sites such as the bones and liver (10 patients), followed by local relapse (7 patients), and one developed relapse in both nodal and primary sites. Only one patient had a node only relapse, and he was offered salvage neck dissection. Of the seven local relapses, five were infield recurrences at the high-risk CTV region (CTVp66), and the other two had relapsed outside the intermediate risk CTV region (CTVp60).

A total of five (6.2%) patients developed second malignancy after a mean period of 17 months—two patients had second primary in the lung and one each in the breast, parotid, and non-Hodgkin's lymphoma. A total of 27 patients (33.3%) died during follow-up. Majority of the deaths (22 patients) were due to disease progression. The other five deaths were due to second malignancy lung, sepsis, lower respiratory tract infections (two), and chronic alcoholic liver disease.

**Figure 1:**
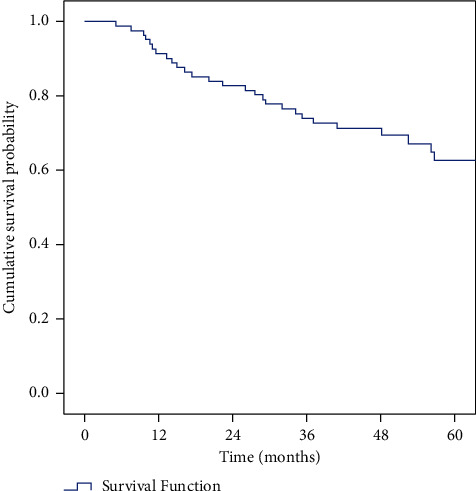
Kaplan–Meier curve showing OS.

**Figure 2:**
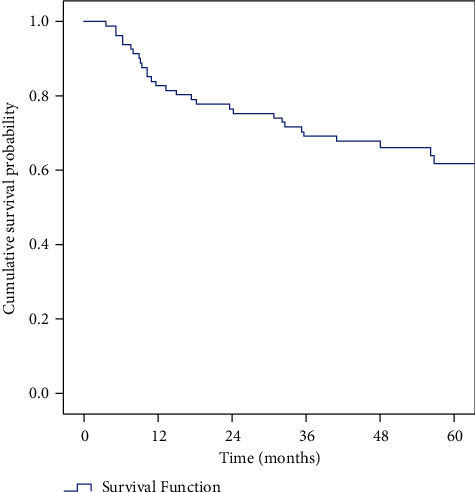
Kaplan–Meier curve showing DFS.

The survival rates are given in Table 3. Stagewise OS and DFS are given in Tables 4 and 5, respectively. (Figures 1 and 2).

The outcome measures (DFS, OS, LRC, and DMFS) were correlated with various patient, tumor, and treatment-related factors and were tested for significance. No factors were found significant on the univariate analysis; hence, the multivariate analysis was not performed in the study.

## 4. Discussion

IMRT in nasopharyngeal carcinoma has resulted in delivering highly conformal radiation dose to the tumor sparing the adjacent organs at risk. Use of chemotherapy in combination with IMRT has been found to provide excellent loco regional control.

A recent meta-analysis had demonstrated that IMRT provides improved long-term tumor control, overall survival, and local control with a lower incidence of late toxicities when compared to 2D RT [9]. A Korean multiinstitutional retrospective study had shown that 5-year overall survival rates were better with 3DCRT and IMRT techniques when compared to 2D radiotherapy techniques [10]. Various other studies also have shown better outcomes and less adverse effects with the use of IMRT [6, 11]. Eventhough there are many benefits for IMRT, it has a potential drawback. An incorrect delineation of the target or normal structures can cause marginal or complete misses, and any small organ motion can cause geometrical error since the dose to target volumes is more conformal with IMRT.

In this retrospective analysis, all the patients were treated with intensity-modulated radiotherapy with the SIB technique. No treatment delayed toxicities were reported, and all patients completed radiotherapy without any interruption.

The 5-year locoregional relapse-free survival for carcinoma nasopharynx treated by IMRT ranges between 70% and 85% as shown in studies by Chen et al. [12] and Sun et al. [13]. In the present study, the loco regional control rate was slightly lower compared to the above studies (LRC at 3 years 74% and at 5 years 62.5%). The patients with local failures were critically evaluated and found that five were infield recurrences at the high-risk CTV region, and majority of patients who relapsed loco regionally had initial *T*3 and *T*4 disease which may have contributed to the above results.

A study by Wang et al. [14] showed that that the 5-year DFS and OS rates of 695 patients treated with the IMRT technique were 69.6% and 77.1%, respectively. Various other studies [10, 15] also have shown 5-year survival rates around 70%. In the present study, the five-year overall survival and five-year disease-free survival was 62.5% and 61.6%, respectively, which is slightly inferior compared to the above major studies.

Various studies have shown that advanced age is a strong and independent predictor of poor disease-free survival and cancer-specific survival in carcinoma nasopharynx [16, 17]. In the present study, patients were stratified into two age groups, age less than 50 and age more than or equal to 50 years, and analyzed for any association with the outcome. A favorable outcome was seen for patients less than 50 years in terms of 3-year distant metastasis-free survival (100% vs. 92%, *p* value = 0.211), disease-free survival (74.5% vs. 70.8%, *p* value = 0.663), and overall survival (78.8% vs. 65%, *p* value = 0.172) when compared to patients with age more than or equal to 50 years.

Studies have shown that the stage of the disease is another important predictor for survival especially for OS and DFS [18, 19]. In the present study, the univariate analysis did not show any significant association with the composite stage (OS: 1, 100%; II, 67%; III, 70.4%; IV, 68.1%; *p* = value, 0.715) and *T* stage of disease(OS: T1, 84.6%; T2, 55.8%; T3, 58.1%; T4, 59.8%; *p* = value 0.553) with respect to survival outcomes. A nonsignificant decrease in 5-year overall survival was observed with increase in the nodal stage (N0, 90.9%; N1, 75.9%; N2, 70%; N3, 63.5%; *p* value - 0.698).

Concurrent chemoradiation is the standard of care in Ca nasopharynx [1]. However, recently, several trials have shown that induction chemotherapy followed by concurrent chemoradiation has better outcomes when compared to concurrent chemoradiation alone [20–25]. In the present study, a favorable outcome was seen in patients who received neoadjuvant chemotherapy when compared to those who did not receive neoadjuvant chemotherapy in terms of OS (68.9% vs. 59.6%, *p* value = 0.838), DMFS (97.5% vs. 82.3%, *p* value = 0.286), and DFS (70.4% vs. 64%, *p* value = 0.885), though they were not statistically significant. Majority of the patients (85.2%) in the present study received concurrent chemotherapy.

The most common site of relapse in nasopharyngeal carcinoma is distant sites [6, 26, 27]. Several studies which evaluated the effectiveness of IMRT have also demonstrated that the most common site of treatment failure was at distant sites [14, 28]. In the present study, 41.6% of failure was at distant sites. Distant metastasis was also a major cause of death. Most patients who developed distant metastasis in our study had either *T*3, *T*4, or *N*3 disease at presentation. Even though IMRT provides excellent locoregional control, it does not control the distant failures.

Compliance to treatment was good in this study. Out of the 42 patients who received neoadjuvant chemotherapy, 37 could tolerate concurrent chemotherapy without interruption. Tolerance to radiation was also good with none of the patients having developed any interruption during the planned course of radiotherapy.

Various authors have reported varying incidence (0.04%–5.3%) of second malignancies in patients with carcinoma nasopharynx [29, 30]. The variations in the incidence of second malignancies may be a function of duration of follow-up or genetic predisposition, techniques of radiotherapy, Epstein–Barr virus, or tobacco and alcohol use. In our study, total of five patients developed second malignancy. Of these, two patients had lung cancer which can be attributed to their smoking habit.

The retrospective study design and the small number of sample size were the major limitations of our study. In addition, dosimetry analysis and evaluation of quality of life were also not included in the present study. We have also not performed the EBV assay for any of the patients. However, in this single institution study, all the patients were treated with uniform radiotherapy schedule, and most of the patients received platinum-based chemotherapy. The same treatment protocol was used for all patients. Majority of the patients had good compliance to the treatment. The follow-up information was available for all patients in the present study.

## 5. Conclusion

In the present study, which shows our initial experience with IMRT, the 5-year survival was slightly lower compared with the other published results. The compliance to RT was good in our study. Majority of relapses seen were distant metastasis. Neoadjuvant chemotherapy is a reasonable option to be explored in the future to prevent distant relapses.

## Figures and Tables

**Table 1:** Baseline characteristics of patients in the study.

Variables		*N* = 81 (%)
Age	<50 years	52 (64.2%)
	>50 years	29 (35.8%)

Gender	Male	55 (67.9%)
	Female	26 (32.1%)

Habits	Yes	17 (221%)
	No	64 (79%)

Comorbid illness	Yes	25 (30.8%)
	No	56 (69.2%)

*T* stage	1	13 (16%)
	2	27 (33.3%)
	3	23 (28.4%)
	4	18 (22.3%)

*N* stage	0	11 (13.6%)
	1	29 (35.8%)
	2	27 (33.3%)
	3	14 (17.3%)

Composite stage	I	2 (2.5%)
	II	19 (23.5%)
	III	31 (38.3%)
	IV	29 (35.8%)

Histology	WHO type 1	3 (3.7%)
	WHO type 2a	0 (0%)
	WHO type 2b	78 (96.3%)

**Table 2:** Distribution of patients according to the sequence of chemotherapy.

Chemotherapy sequencing	No. of patients (%)
Neoadjuvant chemotherapy alone	5 (6.3%)
Neoadjuvant + concurrent	34 (41.9%)
Concurrent + adjuvant	4 (4.9%)
Neoadjuvant + concurrent + adjuvant	3 (3.7%)
Concurrent alone	28 (34.5%)
No chemotherapy	7 (8.7%)

**Table 3:** Five-year LRC, DFS, OS, and DMFS.

Survival	5-year (SE)
Locoregional control	87.5% (3.9%)
Disease-free survival	61.6% (5.8%)
Overall survival	62.5 (6.1%)
Distant metastasis-free survival	87% (3.8%)

**Table 4:** Stagewise 5-year DFS.

Stage (no. of patients)	Survival probability at 5 years (%)	Log-rank	*P* value
I (*n* = 2)	100	4.885	0.180
II (*n* = 19)	61.1		
III (*n* = 31)	56.2		
IV (*n* = 29)	84.8		

**Table 5:** Stagewise 5-year OS.

Stage (*n* = no. of patients)	Survival probability (%)	Log-rank	*P* value
I (*n* = 2)	100	1.359	0.715
II (*n* = 19)	67.0		
III (*n* = 31)	70.4		
IV (*n* = 29)	68.1		

## Data Availability

The excel data sheet containing the data supporting the findings of the study is available from the first author upon request.
